# Osteochondral Tissue Regeneration Using a Tyramine-Modified Bilayered PLGA Scaffold Combined with Articular Chondrocytes in a Porcine Model

**DOI:** 10.3390/ijms20020326

**Published:** 2019-01-15

**Authors:** Tzu-Hsiang Lin, Hsueh-Chun Wang, Wen-Hui Cheng, Horng-Chaung Hsu, Ming-Long Yeh

**Affiliations:** 1 Department of Biomedical Engineering, National Cheng Kung University, 1 University Rd., Tainan 701, Taiwan; jeff.qbmc@yahoo.com.tw (T.-H.L.); whc32002@hotmail.com (H.-C.W.); iloveuk4ever@gmail.com (W.-H.C.); 2 Department of Orthopedics, China Medical University Hospital, 2 Yude Rd., Taichung 40447, Taiwan; d4749@mail.cmuh.org.tw; 3 Medical Device Innovation Center, National Cheng Kung University, 1 University Rd., Tainan 701, Taiwan

**Keywords:** bilayer, chondrocyte, PLGA, tyramine, osteochondral regeneration

## Abstract

Repairing damaged articular cartilage is challenging due to the limited regenerative capacity of hyaline cartilage. In this study, we fabricated a bilayered poly (lactic-co-glycolic acid) (PLGA) scaffold with small (200–300 μm) and large (200–500 μm) pores by salt leaching to stimulate chondrocyte differentiation, cartilage formation, and endochondral ossification. The scaffold surface was treated with tyramine to promote scaffold integration into native tissue. Porcine chondrocytes retained a round shape during differentiation when grown on the small pore size scaffold, and had a fibroblast-like morphology during transdifferentiation in the large pore size scaffold after five days of culture. Tyramine-treated scaffolds with mixed pore sizes seeded with chondrocytes were pressed into three-mm porcine osteochondral defects; tyramine treatment enhanced the adhesion of the small pore size scaffold to osteochondral tissue and increased glycosaminoglycan and collagen type II (Col II) contents, while reducing collagen type X (Col X) production in the cartilage layer. Col X content was higher for scaffolds with a large pore size, which was accompanied by the enhanced generation of subchondral bone. Thus, chondrocytes seeded in tyramine-treated bilayered scaffolds with small and large pores in the upper and lower parts, respectively, can promote osteochondral regeneration and integration for articular cartilage repair.

## 1. Introduction

Osteochondritis dissecans (OCD) is a joint disorder in which cracks form in the subchondral bone that can progress to the overlying articular cartilage. OCD can be caused by genetics, ischemia, and repeated trauma, and is a common cause of knee pain and dysfunction among skeletally immature and young adult patients, athletes, and the elderly [1,2]. Damaged articular cartilage can collapse and create a defect, for which there is a range of treatment options including microfracture [3], mosaicplasty [4,5], and autologous chondrocyte implantation [6]. However, these methods have various problems that remain to be resolved [7,8,9]. Tissue engineering technologies offer a promising treatment approach [10]. Bilayered scaffolds are composed of chondrogenic and osteogenic layers combining cells and/or growth factors that promote the regeneration of specific tissues [11,12]. Two commonly used cell types for osteochondral regeneration are tissue-specific cells and mesenchymal stem cells. The former include chondrocytes and osteoblasts that are used for hyaline cartilage and subchondral bone regeneration, respectively [6,13,14,15]. Chondrocytes dedifferentiate when cultured on a two-dimensional surface, in contrast to cells that maintain their phenotype when cultured in a three-dimensional (3D) gel [16]. Thus, a 3D bioscaffold can provide a suitable culture environment for chondrocytes while preventing their dedifferentiation, making it suitable for cartilage repair.

Osteochondral scaffolds are typically composed of natural materials including fibrin [17], collagen [18,19,20,21], and chitosan [11], but can also include poly(lactide-co-glycolide) (PLGA), which is a synthetic polymer that is widely used in tissue engineering due to its biocompatibility, biodegradability, high porosity, and mechanical strength [22]. The pore size and interconnectivity of PLGA can be easily controlled; therefore, it is a useful material to construct scaffolds with different mechanical properties and degradation rates for the repair of chondral and subchondral bone layers [12,23]. We previously demonstrated that a degradable porous PLGA scaffold creates a temporary space for tissue regeneration and stimulated the repair of cartilage defects [24,25]. We also reported that a porous PLGA scaffold combined with endothelial progenitor cells can repair osteochondral defects in vivo by providing mechanical support and promoting cell recruitment [26].

Controlling the scaffold pore structure is critical for optimal tissue development [27,28,29,30,31,32]. Pore size plays an important role in cell behavior and growth, since it can facilitate cell penetration and migration during cell seeding, as well as nutrient transport and the removal of metabolic substances, and provides a 3D microenvironment that is conducive to cell assembly and differentiation [33]. Scaffolds with a small pore size were shown to induce cell aggregation, increase the cell-specific synthesis of cartilaginous matrix proteins, enhance the chondrocyte phenotype, and promote chondrogenesis in the extracellular matrix around chondrocytes [34,35,36]. Moreover, scaffolds with small mean pore sizes (130–300 μm) were associated with higher rates of chondrocyte differentiation [37]. Highly proliferating chondrocytes and cartilage-specific ECM production were observed on scaffolds with a small pore size of 250–350 μm [38], and 3D scaffolds with a pore size of around 150–300 μm also showed greater chondrocyte growth and chondrogenic differentiation as compared to those with a pore size of 300–500 μm [36]. Additionally, owing to the hierarchical structure of native bone tissue, pore architecture plays a key role in osteogenic differentiation. The minimum pore size for 3D bone regeneration ranges from 100 to >300 μm [39]. A pore size of around 100 μm creates hypoxic conditions that favor osteochondrogenesis over osteogenesis, whereas a larger pore size (>300 μm) directly induces the latter [39]. Pores larger than ~300 μm are essential for vascularization and bone ingrowth, whereas those that are smaller promote osteochondral ossification [40,41,42]. The adhesion and differentiation of osteoblasts was enhanced on scaffolds with a 325-μm pore size after seven days of culture [43]. On the other hand, an in vivo study showed that histological and biochemical scores were optimal for cell-seeded bilayered PLGA scaffolds with 100–200 μm pores in the chondral layer and 300–450 μm pores in the osseous layer [44].

Typical techniques for fabricating PLGA scaffolds include adjusting the surface of grafts for optimal cell–biomaterial interaction or blending materials with mineral particles to adjust for mechanical properties and bioactivity [45,46]. Grafting the hydrophilic active moieties such as carboxyl and amine groups to the surface of PLGA scaffolds by chemical processes can enhance their biological activities. For instance, amine groups can be conjugated by treatment with 1,6-hexanediamine/propanol solution or ethylenediamine (ED) to ensure a positive surface charge [47,48]. ED is a strongly basic diamine that is used with various polymers such as poly(methyl methacrylate), polylactic acid, and PLGA [49,50,51,52,53,54,55,56]. Tyramine is a naturally occurring monoamine derived from the amino acid tyrosine that is abundant in plants and animals. Recent studies have shown that introducing hydroxyphenyl groups into the backbone of naturally occurring polymers such as tyramine, hyaluronic acid, and chitosan promotes covalent bond formation between hydroxyphenyl groups and native collagen [57,58,59,60].

In this study, we speculated that bilayered PLGA scaffolds with a small mean pore size in the upper layer would enhance the chondrocyte phenotype and thus promote the chondrogenic differentiation of chondrocytes for cartilage repair, whereas a large mean pore size in the lower layer would direct chondrocyte hypertrophy toward an osteoblast phenotype for subchondral bone regeneration. Additionally, we hypothesized that the combination of a bilayered PLGA scaffold and tyramine would enhance the integration of regenerated and host osteochondral tissue. Thus, the aim of this study was to investigate the combined effect of pore size and tyramine on osteochondral tissue development in bilayered scaffolds seeded with articular chondrocytes. To this end, we examined the mean pore size of bilayered scaffolds by physical and cytotoxicity assays, and evaluated the potential of tyramine-treated scaffolds seeded with porcine articular chondrocytes to regenerate osteochondral tissue in an ex vivo osteochondral plug by biomechanical, histological, and immunohistochemical analyses.

## 2. Results

### 2.1. Morphology of Bilayered PLGA Scaffolds

**Figure 1:**
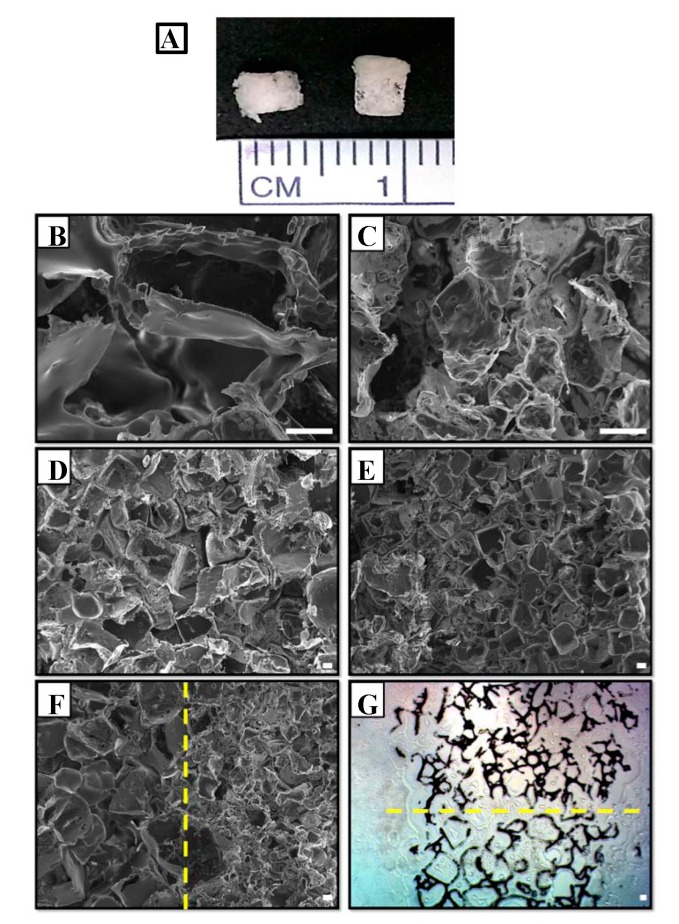
SEM images of bilayered porous poly (lactic-co-glycolic acid) (PLGA) scaffolds. (**A**) Macroscopic view of the scaffold. (**B**–**G**) Microscopic views of the scaffold: high magnification image of large pores (200×) (**B**); high magnification image of small pores (200×) (**C**); low magnification image of large pores (50×) (**D**); low magnification image of small pores (50×) (**E**); bilayered PLGA scaffold (50×) (**F**); and light micrograph of a frozen section of a bilayered PLGA scaffold (**G**). The yellow dotted line in (**F**) and (**G**) represents the boundary between the lower (large pore) and upper (small pore) layers. Scale bar: 100 μm.

A bilayered PLGA scaffold was generated with a height of three mm and diameter of three mm (Figure 1A). There were slight differences in morphology between the upper and lower layers, which had small and large pores, respectively.

### 2.2. Physical Properties of Biphasic PLGA Scaffolds

Compressive modulus and porosity were measured to determine whether a small pore size alters the mechanical properties of the scaffold. There were no differences in these parameters among scaffolds with large, small, and mixed pore sizes (Table 1). A scanning electron microscopy (SEM) analysis of the inner microstructure of the bilayered PLGA scaffold (Figure 1B–F) showed that the pore was approximately 200–300 μm; this upper layer was designed with a smaller pore size to allow chondrocytes to maintain their phenotype and enhance chondrogenesis (Figure 1C,E). The lower layer with a pore size of about 200–500 μm was designed to induce the hypertrophy of chondrocytes and their differentiation into osteoblasts, which are cells in the bone (Figure 1B,D). The two distinct layers were visible in frozen sections (Figure 1G).

### 2.3. Effect of Pore Size of Bilayered PLGA Scaffolds on Chondrocyte Morphology

**Figure 2:**
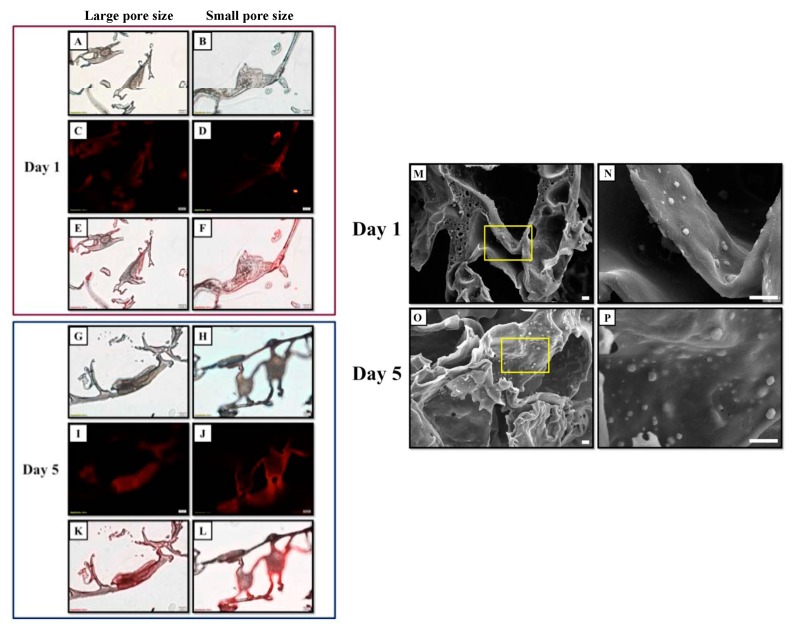
Images of chondrocytes on PLGA scaffolds with different pores sizes after one and five days of culture. The cell membrane was labeled with red fluorescent dye. (**A**,**B**,**G**,**H**) Light micrographs. (**C**,**D**,**I**,**J**) Fluorescence micrographs. (**E**,**F**,**K**,**L**) Merged light and fluorescence micrographs. The red and blue boxes indicate the images of cells on PLGA scaffolds at day one and five, respectively. Scale bar: 20 μm. (**M**–**P**) SEM images of cell-seeded PLGA scaffold with a small pore size: images of cells cultured on PLGA scaffolds on day one (**M**,**N**) and day five (**O**,**P**). The area enclosed by the yellow box is shown enlarged to the right. Scale bar: 10 μm.

When fluorophore-labeled chondrocytes were injected into scaffolds with two different pore sizes, we observed that pore size markedly influenced cell morphology. After one day of culture, chondrocytes had a fibroblast-like appearance in the large pore size scaffold (Figure 2A,C,E), whereas chondrocytes in the small pore size scaffold maintained their original shape (Figure 2B,D,F). The morphology of chondrocytes in large pore size scaffolds became more fibroblast-like after five days of culture (Figure 2G,I,K). Conversely, chondrocytes in the small pore size scaffold maintained a round shape and formed aggregates, which can enhance chondrogenesis (Figure 2H,J,L). SEM analysis confirmed that chondrocytes cultured in small pore size scaffolds had a round morphology and showed greater aggregation after one and five days of culture (Figure 2M–P).

### 2.4. Characteristics of Bilayered PLGA Scaffolds with Surface Modification

#### 2.4.1. Water Contact Angle of Surface-Modified Bilayered PLGA Scaffolds

**Figure 3:**
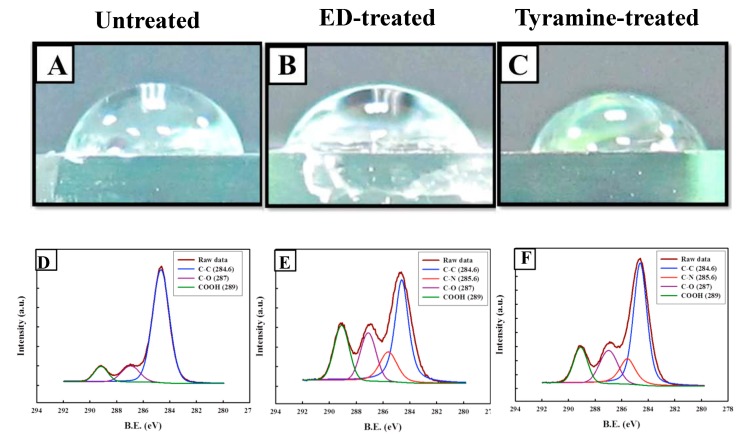
Water contact angle and results of surface analysis by X-ray photoelectron spectroscopy (XPS) of untreated and surface-treated PLGA scaffolds. (**A**,**D**) Untreated scaffolds. (**B**,**E**) Ethylenediamine (ED)-treated scaffolds. (**C**,**F**) Tyramine-treated scaffolds.

Hydrophilicity can affect cell adhesion and growth; therefore, we treated the surface of bilayered PLGA scaffolds with ED or tyramine, and measured the water contact angle to evaluate their hydrophilicity (Figure 3A–C). The water contact angle decreased, while the surface hydrophilicity increased upon surface modification with ED and tyramine; the results showed that the water contact angle of the untreated PLGA group (68.08 ± 1.8°) was significantly higher than that of the ED-treated (57.10 ± 2.14°) (*p* < 0.01) or tyramine-treated (52.58 ± 1.43°) (*p* < 0.01) groups, respectively. Additionally, the water contact angle of the ED-treated group was significantly higher than that of the tyramine-treated group (*p* < 0.01). The rank order of the water contact angle was untreated > ED-treated > tyramine-treated (Table 2).

#### 2.4.2. Chemical Composition of Surface-Modified Bilayered PLGA Scaffolds

The surface composition of PLGA scaffolds after surface modification was evaluated by X-ray photoelectron spectroscopy (XPS). The untreated PLGA surface showed three main peaks corresponding to C–C/C–H (at 284.6 eV), C–O (287 eV), and –COOH (289-eV shift) [61]. The treated PLGA surfaces showed C–N bonding energy located at 285.6 eV; the signal intensity of this peak was stronger in the scaffold treated with tyramine as compared to the ethylenediamine (ED)-treated or the untreated scaffold (Figure 3D–F). The percent composition of the components is shown in Table 3. The X-ray photoelectron (XP) spectra showed that the tyramine-treated PLGA scaffolds had the highest nitrogen content; the nitrogen peak for ED-treated or tyramine-treated PLGA provided evidence for the successful modification of these scaffolds, since it was completely absent in untreated scaffolds.

#### 2.4.3. Biocompatibility of Surface-Modified Bilayered PLGA Scaffolds

**Figure 4:**
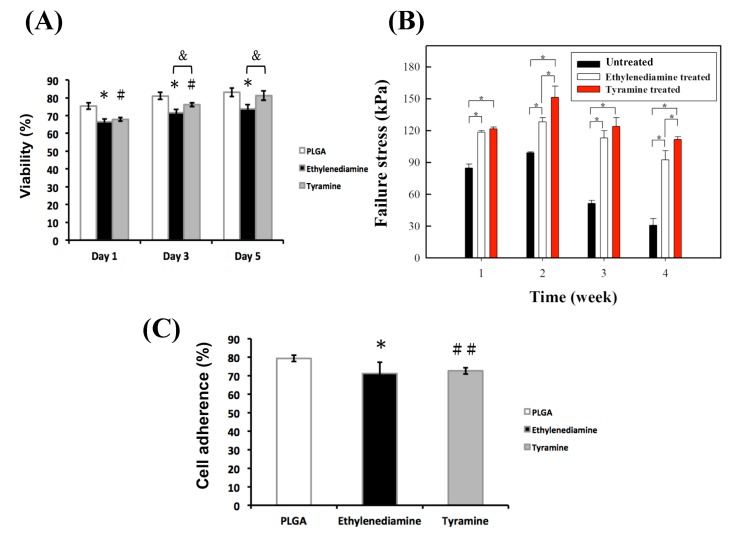
(**A**) Cell viability. Values represent mean ± SD (*n* = 6). * *p* < 0.05 and ^#^ *p* < 0.05: ED-treated or tyramine-treated groups versus the untreated PLGA group, respectively. ^&^ *p* < 0.05: ED-treated group versus the tyramine-treated PLGA group. (**B**) Push-out test results for untreated and surface-treated PLGA scaffolds. Values represent mean ± SD (*n* = 4). * *p* < 0.05. (**C**) Cell adherence of chondrocytes cultured in untreated, ED- or tyramine-treated PLGA scaffolds at day one. Values represent mean ± SD (*n* = 4). * *p* < 0.05 and ^##^ *p* < 0.01: ED- or tyramine-treated groups versus the untreated PLGA group, respectively.

The biocompatibility of the untreated or surface-modified bilayered PLGA scaffolds over one day, three days, and five days was evaluated with the alamarBlue assay using porcine articular chondrocytes (Figure 4A). After one day of culture on the different scaffolds, the viability of untreated, ED-treated, or tyramine-treated groups was 75.4%, 66.5%, and 67.9%, respectively; this increased to 83.1%, 73.6%, and 81.3%, respectively, on day five, demonstrating that untreated PLGA scaffolds have significantly greater biocompatibility than ED-treated (*p* < 0.05) scaffolds, whereas there was no statistically significant difference between the untreated and tyramine-treated groups. Additionally, PLGA scaffolds treated with tyramine showed significantly higher cell viability than those treated with ED (*p* < 0.05) at day five, and are thus safe for use in cartilage regeneration. For the cell adhesion assay, the results showed a significant difference in cell adhesion to untreated and ED-treated PLGA scaffolds after one day of culture (*p* < 0.05) (Figure 4C). Moreover, there was a statistically significant difference in cell adhesion between the untreated and tyramine-treated groups (*p* < 0.01) (Figure 4C). 

#### 2.4.4. Adhesive Strength of the Scaffold–Cartilage Interface

The strength of the scaffold–cartilage interface after one week, two weeks, three weeks, and four weeks of culture was evaluated with the push-out test. Although an adhesive interface developed between each implanted scaffold and native cartilage, major differences were observed between the untreated and surface-treated groups; the latter showed significantly higher (*p* < 0.05) interface strength than the former over the period of examination. The tyramine-treated scaffold showed significantly greater integration than the ED-treated group (*p* < 0.05) at two weeks and four weeks of culture (Figure 4B), which was possibly due to covalent bonding between the hydroxyphenyl groups and native cartilage tissue.

#### 2.4.5. Histological and Immunohistochemical Analyses of Scaffold–Cartilage Constructs in Osteochondral Tissue

**Figure 5:**
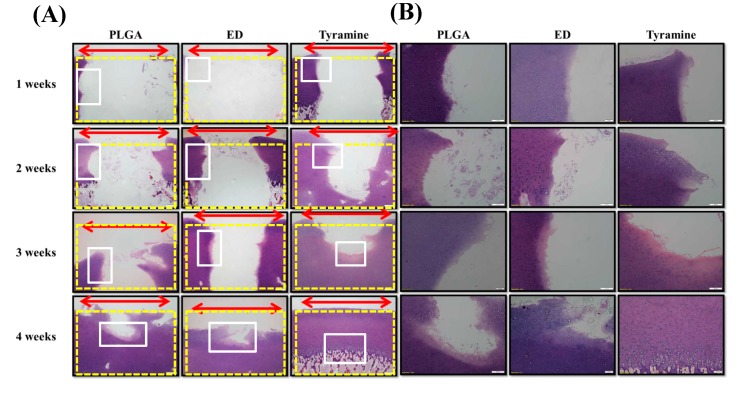
Histological analysis of co-cultured bilayered PLGA scaffold and porcine osteochondral plug by hematoxylin and eosin staining. (**A**) Magnification: 40×; scale bar: 200 μm. (**B**) Magnification: 100×; scale bar: 100 μm. Red double-headed arrows (←→) indicate the defect area after tissue regeneration. Yellow dotted boxes indicate the defect area before tissue regeneration. White boxes indicate that the images of tissue regeneration area were amplified.

**Figure 6:**
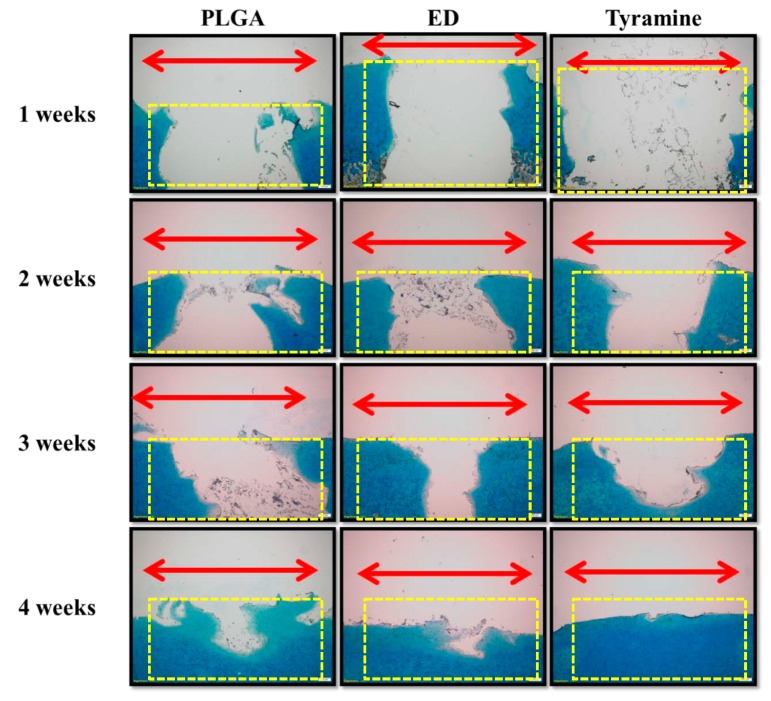
Histological analysis of co-cultured bilayered PLGA scaffold and porcine osteochondral plug by Alcian blue staining. Magnification: 40×; scale bar: 200 μm. Red double-headed arrows (←→) indicate the defect area after tissue regeneration. Yellow dotted boxes indicate the defect area before tissue regeneration.

**Figure 7:**
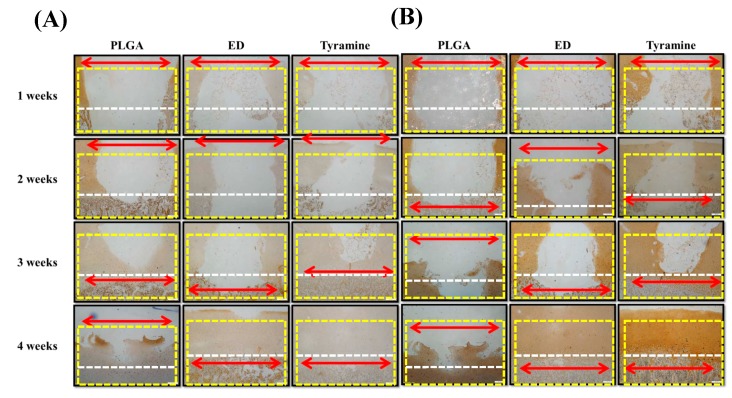
(**A**,**B**) Immunohistochemical detection of collagen type X (Col X) (**A**) and collagen type II (Col II) (**B**) in a co-cultured bilayered PLGA scaffold and porcine osteochondral plug. Magnification: 40×; scale bar: 200 μm. Red double-headed arrows (←→) indicate the defect area after tissue regeneration. Yellow dotted boxes indicate the defect area before tissue regeneration. White dotted lines are used to distinguish the areas for cartilage and subchondral bone.

Histological and immunohistochemical analyses were carried out to observe the integration of surface-treated scaffolds into the osteochondral plug and to evaluate tissue regeneration by these scaffolds after one week, two weeks, three weeks, and four weeks of culture. There was good integration by both the ED-treated and tyramine-treated scaffolds into native osteochondral tissue after four weeks of culture (Figure 5A,B), with regenerated tissue showing complete fusion with adjacent cartilage and bone. In particular, in the tyramine-treated group, reconstructed hyaline cartilage had a normal columnar chondrocyte arrangement, with abundant glycosaminoglycan (GAG) and collagen type II (Col II) and less Col X expression in the cartilage layer four weeks after implantation (Figure 6 and Figure 7A,B). In contrast, the untreated group showed mainly fibrotrophic or hypertrophic cartilage tissue with a disorganized arrangement of cells, as well as reduced GAG and Col II and elevated Col X levels within the defect and integration sites. Chondrocyte hypertrophy is a key event in cartilage calcification and the thickening of subchondral bone, of which Col X is a well-known marker. The tyramine-treated group had higher levels of Col X in both newly formed bone tissue and adjacent subchondral bone after four weeks (Figure 7A) than the other groups. Moreover, a newly synthesized mineral matrix was observed in the surface-treated groups in areas adjacent to the scaffold after four weeks of culture. In the surface-treated groups, the tissue architecture reflected well-integrated subchondral trabecular bone with mature osteocytes. Surprisingly, in the tyramine-treated group, newly grown osseous tissue formed close to native bone, and was indistinguishable from the adjacent host subchondral bone tissue after four weeks of culture, indicating an excellent integration of the implant (Figure 5A,B and Figure 7A).

## 3. Discussion

Repairing osteochondral lesions involving both cartilage and underlying bone remains a major challenge in regenerative medicine. Multilayered scaffold systems have been developed for orthopedic applications [62,63], but these structures require a functional and mechanically stable interface between layers. Our bilayered PLGA scaffolds (Figure 1F) showed a clear interface between cartilage-like and bone-like layers with small (200–300 μm) and large (200–500 μm) pore sizes, respectively, while maintaining overall cohesion.

The current trend in cartilage tissue engineering is to combine cells and synthetic biomaterial scaffolds. Since the composition, architecture, and mechanical properties of biomaterials affect chondrogenesis and cell behavior [64], in this study, we investigated the influence of physical features of bilayered PLGA scaffolds such as pore size on the biological activity and differentiation of seeded chondrocytes. Our results showed that pore size did not directly affect the porosity and compressive modulus of the scaffold (Table 1). The PLGA scaffold was fabricated by a salt-leaching method, and porosity was determined by the weight ratio of salt and a PLGA–chloroform solution.

A tissue engineering scaffold should be biocompatible and mimic both the physical and biological functions of the native extracellular matrix (ECM), which provides a substrate with specific ligands for cell adhesion as well as physical support to cells [65]. Mean pore size is also an important consideration: pores that are too small limit cell migration, leading to the formation of a cell capsule around the scaffold edge that can in turn limit the distribution of nutrients and removal of waste products, resulting in necrotic regions within the construct. On the other hand, when pores are too large, the specific surface area is reduced [66], which decreases the amount of ligand that is available to cells for binding. Additionally, the mean pore size of a scaffold affects cell adhesion and subsequent proliferation, migration, and differentiation. Therefore, it is necessary to maintain a balance between the optimal pore size for cell migration and specific surface area for cell attachment [39,65]. In this study, we constructed a bilayered PLGA scaffold with pores of different sizes. The results of the cellular activity assay showed that chondrocytes cultured in the cartilage layer of the scaffold with a small pore size retained a chondrocyte phenotype, whereas those in the bone layer with large pores were induced to dedifferentiate (Figure 2A–L). Moreover, the former showed increased proliferation and aggregation (Figure 2F,L,M–P), which is an important cue for the expression of chondrocyte markers [67,68]. Thus, the microstructure of the scaffold in the cartilage layer affects the chondrogenic differentiation of chondrocytes in a three-dimensional (3D) environment, thereby influencing ECM accumulation, cell–cell communication, and chondrocyte differentiation. Some studies have shown that chondrocyte phenotype and biosynthetic activity were enhanced in 3D scaffolds containing smaller pores [34,35,36,37,38], which is consistent with our findings. On the other hand, the microarchitecture of bilayered PLGA scaffolds also plays an important role in the osteogenic differentiation of chondrocytes: the cells had a fibroblast-like morphology when cultured in a scaffold with a larger pore size (Figure 2E,K), which may induce initial dedifferentiation during the early stage of chondrocyte transdifferentiation, followed by their redifferentiation into hypertrophic chondrocytes that differentiate into osteoblasts and osteocytes [69]. A similar effect of pore size was observed in the osteogenic differentiation of transgene-expressing mesenchymal stem cells, which was induced to a greater extent in cells grown on a 3D protein scaffold with large (100–300 μm) as compared to small (50–100 μm) pores [70]. Additionally, a large deposit of mineralized tissue was detected in chondrocyte-seeded 3D scaffolds with a larger pore size (300–500 μm) [36]. Another study showed that bone ingrowths predominated in porous 3D scaffold with a pore size of approximately 450 μm [71]. Osteogenesis was also reported to be enhanced by implants with a pore size >300 μm [40,42,72]; larger pores promote osteogenesis by allowing vascularization and high oxygenation.

Cell behavior can vary according to the roughness of the substrate and the geometry of the biomaterial surface [73,74]. However, there have been no direct comparisons of the behavior of chondrocytes on scaffolds with different pore sizes. The results of this study show that a smaller pore size (200–300 μm) is more suitable for chondrocyte than for osteocyte growth, since there are no obvious blood vessels in the cartilage layer (Figure 2F,L,M–P and Figure 5A). We also found that larger pores (200–500 μm) enhanced hypertrophic chondrocyte differentiation for subsequent osteogenesis in the bone layer of the scaffold (Figure 2E,K and Figure 7A).

A number of studies have examined the effect of the physical structure of biomaterial scaffolds on chondrogenesis [75,76,77,78]. One study demonstrated that chondrocytes cultured on two poly(l-lactide) fibrous scaffolds with micrometer or nanometer diameter fibers exhibit distinct biological activities [78]. The nanofibrous scaffold culture favors a cartilage phenotype including the chondrocyte morphology and the production of hyaline cartilage-specific ECM markers. Thus, the phenotype of chondrocytes cultured on an engineered fibrous matrix can be regulated by altering the fiber size. However, our results showed that the upper layer of the scaffold with 200–300 μm pores was more effective in maintaining the chondrocyte phenotype than the lower layer with 200–500 μm pores, although this is larger than the spaces between the aforementioned fibers. One possible explanation for this result is that the structure of the fiber scaffold differs from that of porous sponges, which produces distinct cell behaviors. The chondrocytes were spanned between or aggregated on fibers according to the fiber diameter, in contrast to porous PLGA sponges, in which chondrocytes directly attached to the scaffold surface without being fully flattened.

The mechanism of chondrogenesis in chondrocytes is similar to that in mesenchymal cells, but one difference is that chondrocytes that maintain their phenotype are unlikely to become hypertrophic. In the present study, primary cultured chondrocytes could aggregate and proliferate in small pore size scaffolds, suggesting that a pore size of 200–300 μm not only maintains the chondrocyte phenotype, but also promotes chondrogenesis (Figure 2F,L–P).

Scaffold integration is another important factor for cartilage repair; a failure of this process concentrates stress at implant boundaries, predisposing both the surrounding native tissue and the scaffold to further degeneration [79,80]. Commercially available fibrin-based tissue glue for surgical purposes is typically used to increase adhesion at the boundary. Despite being non-toxic, these glues rapidly degrade, especially in the presence of increasing numbers of chondrocytes [81]. Previous studies have shown that chitosan-based adhesive hydrogels are effective for initial fixation, but are slow to gel and are mechanically fragile [82]. Thus, the ability of a scaffold to integrate into native tissue is a major determinant of successful tissue regeneration using cell-based scaffolds.

The covalent immobilization of bioactive compounds onto functionalized polymer surfaces has been used in the fields of biomedicine, textiles, and microelectronics; compounds with bulk properties that match the mechanical property or degradability of the polymer are typically used [83]. Most commercial polymers, including PLGA, have an inert surface. Surface functionalization can promote the integration of osteochondral plugs, and is achieved by wetting, silane monolayer assembly, ionized gas treatment, etc. Treatment with ionized gas such as plasma can promote cell adhesion. However, although it is convenient and does not alter the properties of the polymer, plasma is inappropriate for PLGA scaffolds, since it can only penetrate a few nanometers from the surface, resulting in uneven functionalization. Therefore, we used a wet chemical surface modification approach in this study, whereby the PLGA was treated with liquid reagent to generate reactive functional groups on the material surface. This method does not require specialized equipment, can be carried out in most laboratories, and enables a greater penetration of porous 3D substrates than plasma and other energy-based surface modification techniques [83]. In this study, PLGA scaffolds were treated with ED and tyramine solutions. The PLGA surface was activated by either base hydrolysis or aminolysis. Tyramine was dissolved in sodium hydrogen base solution, and could thus be activated by the hydrolysis of PLGA functional groups, as opposed to the ED-induced breakdown of PLGA surface functional groups through aminolysis. The XPS results showed that tyramine-treated scaffolds had a higher surface content of amine groups than those treated with ED (Figure 3B and Table 3), which can explain their superior integration into native tissue (Figure 5).

The water contact angle can be used to measure surface hydrophilicity; as a surface becomes more oxidized or has more ionizable groups, hydrogen bonding with water is facilitated, and the water droplet spreads along the hydrophilic surface, lowering the contact angle [83]. Small contact angles (<90°) correspond to high hydrophilicity, while large contact angles (>90°) correspond to low hydrophilicity. In this study, all of the PLGA groups showed small contact angles; however, the contact angle of the ED-treated or tyramine-treated groups was different from that of the untreated scaffold (Figure 3A–C, and Table 2), suggesting that the change in the amine content of the scaffold surface caused by ED or tyramine treatment was sufficient to alter the hydrophilicity.

Enzymatic treatment is an efficient way to improve scaffold and tissue integration; a previous study demonstrated that this could improve the bonding strength and promote articular cartilage repair [84]. Enzymatic treatment may partially break down the ECM surrounding wound edge chondrocytes, thus freeing these cells to migrate [85] and repair the defect [84]. Hydrogen peroxide not only serves as a reactant that binds tyramine to native tissue, it also degrades superficial cartilage ECM to allow chondrocytes to migrate toward the scaffold. In the present study, we observed tissue regeneration in the PLGA scaffolds after four weeks; however, the effect was greater in the tyramine-treated group than in the other two groups, as evidenced by the smooth articular surface, mature chondrocytes, organized arrangement of cells, a hyaline cartilaginous phenotype, GAG enrichment, and lack of inflammation. Moreover, Col II-positive regenerated tissue showed good integration into the surrounding adjacent cartilage, and was anchored to the newly generated lamellar bones (Figure 5, Figure 6 and Figure 7).

To mimic the in vivo situation, we used an in vitro scaffold/native osteochondral co-culture system as an ex vivo platform to investigate the effects of the microstructure and surface modification of bilayered PLGA scaffolds on osteochondral repair. Previous studies have shown that the co-culture of a bilayered cell-seeded scaffold placed in a native bovine osteochondral implant for up to eight weeks resulted in the integration of osteochondral tissue regenerated from the seeded cells into the surrounding cartilage and bone, with the adhesion increasing over time [86]. Another study found that a macroporous polyvinyl alcohol/osteochondral plug co-culture system pretreated with collagenase prior to implantation formed a stable interface with articular cartilage, demonstrating its potential application to the repair of focal cartilage defects [87]. Similarly, in the present study, regenerated tissue in the hydrogen peroxide/tyramine-treated group showed greater integration and higher bond strength with the surroundings than other groups (Figure 4B and Figure 5), which was in accordance with previous studies [86,87].

The objective of the present study was to determine the optimal pore size of bilayered PLGA scaffolds to improve chondrocyte chondrogenesis and enhance hypertrophic chondrocyte differentiation to facilitate osteochondral repair. We designed an integrated scaffold with different pore sizes, but did not use an induction medium. Since the implantation of composite chondrocytes and bilayered PLGA scaffold constructs into porcine osteochondral defects may enhance chondrocyte differentiation into tissue, it can be supposed that differentiated chondrocytes seeded in both layers of the scaffold can spontaneously maintain the chondrocyte phenotype or transdifferentiate into osteoblasts without external induction.

This study had several limitations. Firstly, the mechanistic basis for the effect of the PLGA scaffold microstructure on chondrocyte differentiation is unclear. Secondly, since the follow-up period after ex vivo implantation was only four weeks, the long-term outcome of the regenerated tissue in terms of mechanical properties and specific tissue formation is not known. Further optimization of bilayered scaffolds and additional studies in larger animals are necessary in order to recapitulate the actual conditions in human synovial joints.

## 4. Materials and Methods

### 4.1. Fabrication of Bilayered PLGA Scaffold

PLGA (lactide/glycolide ratio of 85/15, molecular weight: 50–75 kDa) scaffolds of two different pore sizes were synthesized using a solvent salt-leaching technique [25]. Briefly, four mL of 20% *(weight/volume)* PLGA chloroform solution was mixed with 3.6 g of NaCl particles (~300 μm in diameter) that were sieved and used as a porogen for the lower layer of the scaffold. The upper layer had the same composition of PLGA solution as the lower layer, but the NaCl grain diameter was ~100 μm. The layer with the larger porogen size was poured into a three-mm cylinder mold; soon after, the small grain size NaCl PLGA solution was poured into the same mold. The bilayered structure was lyophilized at −20 °C for one day to remove the chloroform, yielding PLGA sponges that were immersed in deionized water to dissolve the salt particles. A bilayered porous PLGA scaffold was fabricated with a diameter of three mm, height of three mm, and upper layer thickness of about 1.5 mm.

### 4.2. Physical Properties of Bilayered Scaffold

The morphology of the bilayered scaffold was evaluated by SEM; the pore size was measured from the images using ImageJ software (National Institutes of Health, Bethesda, MD, USA), and porosity (*n* = five per group) was calculated as the difference between bulk and true density according to Formula 1:

(1 − ρt/ρ0) × 100% (Formula 1)(1)

where ρt is the density of the porous PLGA bilayered scaffold, and ρ0 is the standard PLGA density according to the manufacturer (1.3 g/mL) (Sigma-Aldrich, St. Louis, MO, USA). The compressive modulus of the scaffold (*n* = six per group) was measured in a wetted, unconfined state using a material testing system (LRX5K, Lloyd Instruments, U.K.) at a compression rate of one mm/min up to 20 N, and was determined as the initial tangent region of the stress–strain curve [25].

### 4.3. Chondrocyte Isolation

Primary porcine chondrocytes were isolated from three to four-month-old swine. Two porcine femurs were obtained from Chi-Mei Animal Center, Tainan, Taiwan, less than two hours after sacrifice. Cartilage tissue was obtained from the trochlear groove and femoral condyles articular joint and minced into small fragments that were first washed with sterile phosphate-buffered saline (PBS) containing 1% antibiotic–antimycotic solution (10,000 units/mL of penicillin, 10,000 μg/mL of streptomycin, and 25 μg/mL of Fungizone™) (Gibco BRL, Gaithersburg, MD, USA) and 0.1% amphotericin B (Gibco BRL, Gaithersburg, MD, USA). The ECM of cartilage fragments was digested by incubation with collagenase type II (1 mg/mL) (Sigma-Aldrich, St. Louis, MO, USA) for eight hours at 37 °C, and chondrocytes were resuspended in Dulbecco’s modified Eagle’s medium (DMEM)/F12 medium supplemented with 10% fetal bovine serum and 1% antibiotic–antimycotic solution. After digestion, chondrocytes were collected using a 100-μm cell strainer and maintained in DMEM/F12 culture medium.

### 4.4. Chondrocyte Appearance in Bilayered PLGA Scaffold

The cell membrane of isolated chondrocytes was stained with the red fluorescent dye PKH26 using a commercial kit (Sigma-Aldrich, St. Louis, MO, USA). The labeled chondrocytes were loaded into a syringe with a 22-G needle at a concentration of 4 × 10^6^ cell/mL and injected into scaffolds with different pore sizes. The final cell density was 6 × 10^4^/scaffold. Fluorescently labeled samples were harvested one and five days later, fixed overnight in 4% paraformaldehyde solution, embedded in optimal cutting temperature compound (OCT compound) (Sakura-Finetek, Torrance, CA, USA), and stored at −20 °C. The samples were cut at a thickness of five μm, and observed under an inverted fluorescence microscope (Olympus, Tokyo, Japan). SEM samples were prepared according to standard methods, and data were collected at the same time points.

### 4.5. Surface Modification of Bilayered PLGA Scaffold

ED (99.8%; J.T. Baker Chemical Company, Phillipsburg, NJ, USA) was diluted to 25% *v*/*v* with deionized water before use. The fabricated bilayered PLGA scaffold was immersed in ED solution for one minute, and then washed with PBS to obtain ED-modified scaffolds. Tyramine (Sigma-Aldrich, St. Louis, MO, USA) was dissolved in 0.05 M of sodium hydroxide to a final concentration of two weight (wt)%; the PLGA scaffold was immersed in tyramine solution for one hour, and then washed three times with PBS. Untreated bilayered scaffolds served as controls.

### 4.6. Measurement of Water Contact Angle of Surface-Modified Bilayered Scaffold

The hydrophilicity of the ED-treated or tyramine-treated PLGA scaffolds and untreated PLGA scaffolds was measured by water contact angle analysis. Before the test, PLGA was coated on the surface of a pre-cleaned piece of quartz glass. ED and tyramine were coated on separate pieces of quartz glass containing PLGA (*n* = eight per group). Briefly, the samples were placed on the testing plate and kept smooth. Subsequently, 0.01 mL of distilled water was dropped slowly onto the surface of the dry scaffold film. Images of the water droplet on the substrate were acquired with a digital camera (DP70; Olympus, Tokyo, Japan) in the testing system after the droplet was stable. Then, the contact angle was measured with Image J software (https://imagej.nih.gov/ij/).

### 4.7. XPS Analysis of Surface-Modified Bilayered Scaffold

We analyzed the chemical composition of the ED-treated or tyramine-treated and untreated bilayered PLGA scaffolds by XPS (Thermo Fisher Scientific, Waltham, MA, USA). The spectra were recorded using Mg Kα radiation at 0–1000 eV with a 150-W power supply at the anode.

### 4.8. Viability and Cell Adherence in Surface-Modified Bilayered Scaffold

Porcine chondrocytes were seeded on untreated or surface-treated bilayered scaffolds. After one day, three days, and five days, the scaffold (*n* = six per group) was examined with alamarBlue assay (Thermo Fisher Scientific, Waltham, MA, USA) to assess cell proliferation. The reactive solution was added to DMEM/F12 cell culture medium at a 1:10 ratio; the cell-seeded scaffold was incubated in the mixture for two hours at 37 °C and 5% CO_2_. A 100-μL volume of supernatant solution was transferred to a 96-well plate, and cell viability was determined by measuring the absorbance at 590–620 nm on a microplate reader (BioTek, Winooski, VT, USA). For cell adherence tests (*n* = four per group), one day after cells adhered to the untreated or surface-treated PLGA scaffolds, cells/scaffold constructs were rinsed and removed from the 24-well plates. The number of unattached viable cells inside the wells was counted and compared with the control (24-well plate seeded cells without any scaffold) to obtain the number of viable cells attached to each scaffold within one day. The CellTiter 96^®^ AQueous One Solution Cell Proliferation Assay (Promega, Madison, WI, USA), which is a colorimetric method for determining the number of viable cells in culture, was used to obtain the cell numbers. After one day of culture, a freshly prepared 3-(4,5-dimethylthiazol-2-yl)-2,5-diphenyltetrazolium bromide (MTS) reaction mixture diluted in culture medium at a volume ratio of 1:5 (MTS:medium) was added to the wells containing the cells/scaffold constructs and then incubated at 37 ℃ under 5% CO_2_ for an additional four hours. Next, 100 μL of the converted MTS that was released into medium from each well was transferred to 96-well plates, and the absorbance at 490 nm was read with a microplate reader. The cell adherence of chondrocytes was calculated using the following formula:

Cell adherence (%) = [1− (Cell number unattached to scaffold/Control cell number inside wells)] × 100% (Formula 2)(2)

### 4.9. Preparation of Osteochondral Host Tissue Plug and Scaffold–Cartilage Constructs

**Figure 8:**
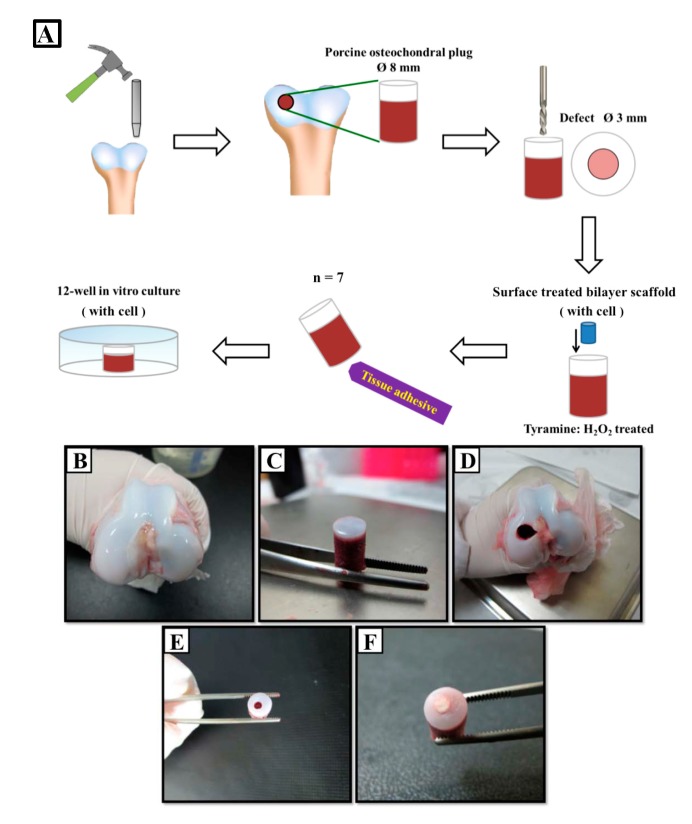
Fabrication of osteochondral host tissue plug and ex vivo co-culture of plug tissue and cell-seeded surface-treated scaffold. (**A**) Schematic illustration of porcine plug manufacture and co-culture of plug tissue and cell-seeded, surface-treated scaffold. Thick white arrows indicate the processes of osteochondral plug manufacture and the cell-seeded surface-treated scaffold implantation; thin black arrow indicates the implantation of cell-seeded surface-treated scaffold in porcine plug; the blue cylinder indicates the cell-seeded surface-treated scaffold. (**B**) Femoral condyles of porcine knee joints. (**C**) An osteochondral tissue plug was created using an eight-mm diameter hole driller. (**D**) Porcine femur after plug removal. (**E**) A three-mm defect was drilled into the osteochondral plug. (**F**) A three-mm cell-seeded bilayered PLGA scaffold was pressed into the defect.

Animal procedures were approved on 9 February 2017 by the Animal Care and Use Committee of National Cheng Kung University (No. 106163), and were performed using the aseptic technique. Cartilage tissue plugs (eight-mm diameter) were cored from the trochlear groove and femoral condyles of porcine knee joints. Defects (three-mm diameter) were created using a sterile drill. The osteochondral plug was pretreated with 3% hydrogen peroxide solution for one minute to expose the collagen functional group. Fabricated scaffolds were cleaned with ethanol and PBS. A 15-μL volume (6 × 10^4^ cells/15 μL) of chondrocyte suspension was loaded onto the top surface of the scaffold and allowed to penetrate the pre-wetted scaffold. The cell–scaffold constructs were then incubated at 37 °C and 5% CO_2_ for four hours to promote cell adhesion. The osteochondral plug was washed with PBS, and cartilage annuli were glued to a 12-well tissue culture plate using a tissue adhesive. Cell scaffolds were pressed into the cartilage plug defect, and two to three mL of DMEM/F12 containing 1% antibiotic–antimycotic and 0.04% amphotericin B were added to completely cover the scaffold–cartilage construct, followed by incubation at 37 °C and 5% CO_2_ (Figure 8) for one week, two weeks, three weeks, and four weeks. The maximum interface strength of the constructs was evaluated with the push-out test (*n* = four per group). Intact constructs (*n* = three per group) that were not used for push-out testing were subjected to histological and immunohistochemical analyses at the same time points.

### 4.10. Biomechanical Analysis of Scaffold–Cartilage Construct

Scaffold–cartilage interface strength was evaluated with the push-out test. Constructs (*n* = four per group) were placed on a material testing machine (LRX5K, Lloyd Instruments, UK) equipped with a 50-N load cell. The test was performed for five minutes with a customized stainless steel stamp with a 2.5-mm diameter. The stamp was advanced through the specimen at a speed of 10 μm/s [88]. A rod indenter was designed that was smaller than the scaffold to prevent misalignment, and both the construct and stamp were carefully aligned before the test. During the experiment, the output values were stored on a laptop computer. For each construct, the peak load-to-failure (maximum observed load) was used to calculate the interface stress-to-failure (maximum load normalized to interface area) to estimate interfacial strength using Formula (3):

Maximum shear stress (τ_Max_) = maximum observed load/2πrH (Formula 3) (3)

where r is the scaffold radius, and H is scaffold thickness.

### 4.11. Histological and Immunohistochemical Analyses

Samples (*n* = three per group) for histological analysis were prepared by the Chi-Mei Medical Center Department of Pathology. Harvested bone tissue plugs were fixed in 10% formalin, dehydrated in a graded series of ethanol, decalcified, infiltrated, and embedded in paraffin. The samples were cut into sections at a thickness of five μm that were stained with hematoxylin and eosin to assess cell morphology and tissue regeneration and with Alcian blue to assess GAG content. The samples were observed under a light microscope (BX51; Olympus, Tokyo, Japan). Images were acquired using a digital camera (DP70; Olympus, Tokyo, Japan). To determine the Col X (hypertrophic cartilage) and Col II (hyaline cartilage) contents of regenerated tissue, the sections were evaluated by immunohistochemistry with primary antibodies (Col X: 14-9771-82, Thermo Fisher Scientific, Waltham, MA, USA; Col II: bs-0709R, Bioss, Woburn, MA, USA) used at 1:100 dilution and a rabbit/mouse horseradish peroxidase/diaminobenzidine polymer detection kit (BioSB, Santa Barbara, CA, USA).

### 4.12. Statistical Analysis

Data are shown as mean ± standard deviation. Statistical analyses were performed using SPSS v.17.0 software (https://www.ibm.com/analytics/spss-statistics-software). Push-out test results were first analyzed with the Kruskal–Wallis test [89], followed by the Mann–Whitney U test [90]. *p* < 0.05 was considered statistically significant.

## 5. Conclusions

This study examined the effect of pore size and the surface modification of integrated bilayered PLGA scaffolds on tissue regeneration in porcine osteochondral defects. Our results demonstrate that chondrocytes maintain their phenotype and exhibit chondrogenic differentiation in the chondral layer, and undergo dedifferentiation in the early stage of transdifferentiation in the osseous layer for osteoblast generation. Tyramine treatment of the bilayered scaffolds seeded with allogenic chondrocytes in the chondral and osseous layers promoted tissue integration and the regeneration of both articular cartilage and subchondral bone. Pore sizes of 200–300 μm in the chondral layer and 200–500 μm in the osseous layer yielded optimal results. These findings highlight the importance of scaffold design for osteochondral tissue engineering and regenerative medicine.

## Figures and Tables

**Table 1:** Compressive modulus and porosity of PLGA scaffolds with different pore sizes. Values represent mean ± SD (*n* = six per group for compressive modulus; *n* = five per group for porosity analysis).

Pore Type	Compressive Modulus (MPa)	Porosity (%)
Large pores	0.517 ± 0.01	88.807 ± 1.29
Small pores	0.516 ± 0.01	89.532 ± 1.38
Mixed sized pores	0.517 ± 0.01	88.997 ± 1.28

**Table 2:** Water contact angle of surface-treated bilayered PLGA scaffolds. Values represent mean ± SD (*n* = 8). ** *p* < 0.01 versus the untreated PLGA group; ^##^ *p* < 0.01: ED-treated group versus the tyramine-treated group.

Contact Angle Analysis	Untreated PLGA	Ethylenediamine-Treated PLGA	Tyramine-Treated PLGA
Contact angle (°)	68.08 ± 1.8°	** 57.10 ± 2.14°	**^,^^##^ 52.58 ± 1.43°

**Table 3:** X-ray photoelectron spectroscopy analysis of surface element composition of surface-treated bilayered PLGA scaffolds.

Surface Treatment	Atom	Atomic Percentage (%)
Untreated	C	76.53
	O	22.60
	N	0.87
Ethylenediamine-treated	C	69.26
	O	29.50
	N	1.24
Tyramine-treated	C	71.54
	O	26.89
	N	1.57

## Abbreviations

3D	Three-dimensional
Col II	Collagen type II
Col X	Collagen type X
ECM	Extracellular matrix
ED	Ethylenediamine
GAG	Glycosaminoglycan
OCD	Osteochondritis dissecans
PBS	Phosphate-buffered saline
PLGA	Poly(lactic-co-glycolic acid)
SEM	Scanning electron microscopy
XPS	X-ray photoelectron spectroscopy
